# A systematic *in silico* report on iron and zinc proteome of *Zea mays*

**DOI:** 10.3389/fpls.2023.1166720

**Published:** 2023-08-17

**Authors:** Ankita Sharma, Dixit Sharma, Shailender Kumar Verma

**Affiliations:** ^1^ Centre for Computational Biology and Bioinformatics, School of Life Sciences, Central University of Himachal Pradesh, District Kangra, Himachal Pradesh, India; ^2^ Department of Environmental Studies, University of Delhi, Delhi, India

**Keywords:** *Zea mays*, iron, zinc, *in silico*, crop improvement

## Abstract

*Zea mays* is an essential staple food crop across the globe. Maize contains macro and micronutrients but is limited in essential mineral micronutrients such as Fe and Zn. Worldwide, serious health concerns have risen due to the deficiencies of essential nutrients in human diets, which rigorously jeopardizes economic development. In the present study, the systematic *in silico* approach has been used to predict Fe and Zn binding proteins from the whole proteome of maize. A total of 356 and 546 putative proteins have been predicted, which contain sequence and structural motifs for Fe and Zn ions, respectively. Furthermore, the functional annotation of these predicted proteins, based on their domains, subcellular localization, gene ontology, and literature support, showed their roles in distinct cellular and biological processes, such as metabolism, gene expression and regulation, transport, stress response, protein folding, and proteolysis. The versatile roles of these shortlisted putative Fe and Zn binding proteins of maize could be used to manipulate many facets of maize physiology. Moreover, in the future, the predicted Fe and Zn binding proteins may act as relevant, novel, and economical markers for various crop improvement programs.

## 1 Introduction

Good nutrition is the source of good health. Good nutrition includes a balanced diet that fulfills our body’s needs for energy, carbohydrates, proteins, vitamins, and mineral micronutrients. Metal micronutrients are essential elements for all organisms as are involved in diverse biological and cellular functions (Crichton, 2020). Humans obtain them from their diet, which primarily relies on plants. In the present scenario, a severe threat to human nutrition may occur in many countries as the deficiencies of essential micronutrients in crop plants are continuously rising and becoming a more serious problem. The main reasons behind this threat are changing climate conditions such as the elevation in temperature, CO_2_, salinity, and drought stresses which directly or indirectly influence nutrient availability (Moreno-Jiménez et al., 2019; Soares et al., 2019). Out of the 7 billion population of the world, more than 2 billion are suffering from micronutrient deficiencies, particularly iron (Fe), zinc (Zn), iodine, and vitamins ((IFPRI), 2016).

Fe plays a critical role in life-sustaining processes like DNA synthesis, respiration, photosynthesis, hormone synthesis and regulation, etc. (Zhang, 2014; Rout and Sahoo, 2015). Fe is not rapidly available in plants due to the low solubility of its abundant ferric (Fe^3+^) form in aerobic environments. Plants use various strategies to convert the Fe^3+^ form into ferrous (Fe^2+^) for their bioavailability and uptake (Morrissey and Guerinot, 2009). The deficiency of this critical element in crop plants led to a reduction in vegetative growth, chlorosis, poor seed quality, and poor yield (Kobayashi and Nishizawa, 2014). WHO estimates that the Fe deficiency in humans is the most prevalent deficiency affecting approximately 3.7 billion people worldwide. The global burden of disease study, 2019 indicates that the global age-standardized point prevalence rates for anemia and years lived with disability (YLDs) were 23,176.2 and 672.4, respectively (Stevens et al., 2022). The most vulnerable groups to Fe deficiency and anemia are pregnant and lactating women, preschool children, and adolescents (Safiri et al., 2021).

Zn is also an essential metal micronutrient cofactor of many enzymes involved in the growth and development of plants and animals. A deficiency of Zn leads to chlorosis, stunted growth, and reduced tiller, leaves, and grain size which results in overall yield reduction (Wissuwa et al., 2006). Worldwide, approximately 17.3% of people are at risk of inadequate Zn intake (Wessells and Brown, 2012). Zn deficiency affects approximately 1/3 of the human population, principally children and women, causing health problems like growth retardation, impaired immune system, diarrhea, eye and skin lesions, weight loss, and mental lethargy (King et al., 2015). Therefore, the significant challenge for the scientific community is to feed the increasing population and provide nutrient-rich food to the masses.

Cereal crops are the world’s paramount food grains rich in three macronutrients (carbohydrates, proteins, and fats) but have suboptimal micronutrients, mainly, Fe and Zn (Borrill et al., 2014). *Zea mays* (Maize) is the world’s third most important staple food crop (Nuss and Tanumihardjo, 2010). The popularity of the maize crop is due to its diverse functionality and multiple uses in human and livestock consumption (Murdia et al., 2016; Shah et al., 2016; Huma et al., 2019). Maize provides both macro and micronutrients vital for various metabolic activities in humans, but, unfortunately, the quantity of some essential micronutrients (lysine, tryptophan, vitamin-B, C, Fe, Zn, and iodine) are ill-balanced (Nuss and Tanumihardjo, 2010; Shah et al., 2016). To combat micronutrient deficiencies in developing countries, food diversification, pharmaceutical supplementation, and post-harvest food fortification interventions have been emphasized. However, only some of these interventions were found to be sustainable.

Biofortification is a cost-effective, sustainable approach for enriching agricultural crops. The biofortification-based conventional breeding and genetic-engineering approaches enhance the micronutrient level in various staple foods (Yunta et al., 2013; Goudia and Hash, 2015). These biofortification-based conventional breeding methods have met with only marginal success, resulting in two-three fold increase in the micronutrient content, which needs to be increased to reach the required level. Various efforts have also been made for the enrichment of Fe and Zn in maize. A transgenic maize plant variety constructed by Drakakaki et al. (2005) co-expresses recombinant soybean ferritin and Aspergillus phytase, having high enzymatic activities to degrade more endogenous phytic acid and significantly raising the uptake of bio-available iron in its endosperm (Drakakaki et al., 2005). Single nucleotide polymorphisms (SNPs) are the markers of choice in genetic analysis and are effectively used in agricultural breeding programs (Gupta et al., 2001). Hindu et al. (2018) conducted a genome-wide association study on 923 inbred maize lines using high-density SNPs to detect the marker-trait associated with high-kernel Zn and Fe in maize and suggested them as precursors to marker-based breeding for the biofortification of the maize (Hindu et al., 2018). Earlier, a translational fusion of maize globin with green fluorescent protein under transcriptional control of the maize endosperm specific 27kDa γ-zein promoter was also done to construct a transformed variety of maize (Bodnar et al., 2013). Expression of this transformed maize grain was evaluated in caco-based cell lines result of which indicate that the transformed variety have bioavailability similar to that of untransformed seeds (Bodnar et al., 2013).

Currently, advancements in the field of omics offered the use of oligo-directed mutagenesis, RNA-directed DNA methylation, gene editing toolkits and availability of completely sequenced genomes of staple foods which open up new rooms for biofortification (Akhtar et al., 2018). Further, the proteomic approaches act as excellent tools to detect micronutrient deficiency and toxicity (López-Millán et al., 2013; Ali et al., 2020; Prusty et al., 2022). Most of the current proteomic studies are focused on the detection of quantitative changes and rely on comparative approaches using two-dimensional gel electrophoresis (2-DE) followed by various mass spectrometry (MS) analyses. The changes under iron deficiency/sufficiency in the root proteome profiles of *Solanum lycopersicum*, *Cucumis sativus*, *Medicago truncatula*, and *Beta vulgaris* have been studied earlier using various proteomic techniques (Li et al., 2008; Donnini et al., 2010; Rellán-Álvarez et al., 2010; Rodriguez-Celma et al., 2011). The proteomic analysis of the sub-proteome such as isolated organelles, phloem saps, and plasma membrane in nutrient-deficit plants is also becoming popular at present time (Laganowsky et al., 2009; Lan et al., 2011; Hopff et al., 2013; Lattanzio et al., 2013). Proteomic studies have been published earlier on the Fe-deficient root and thylakoid proteome of *Arabidopsis thaliana* (Laganowsky et al., 2009; Lan et al., 2011). The plasma-membrane proteome of maize under Fe-deficient and excess conditions has also been studied earlier using nano-liquid chromatography-tandem mass spectrometry (LC-MS/MS), and the study indicated that the proteins involved in signaling and transport are abundantly changed under these conditions (Hopff et al., 2013). Moreover, the accessibility of bioinformatics techniques makes an *in silico* approach realistic for identifying candidate metalloproteins from the complete proteome of an organism (Sharma et al., 2017; Verma et al., 2017; Sharma et al., 2018; Sharma et al., 2019a; Sharma et al., 2019b). Hence, the proposed study here will be designed to explore the putative Fe and Zn binding proteins from the complete proteome of *Zea mays*, which could prove to be a better-starting material, novel, economical, and reliable marker to expedite bioavailability and biofortification as well as other crop improvement programs worldwide.

## 2 Materials and methods

### 2.1 Data extraction

The whole proteome of *Zea mays* (Maize) was retrieved from the RefSeq ftp server of the National Centre for Biotechnology Information (NCBI). Iron binding and Zinc binding keyword searches were done in the Uniprot database to construct Fe and Zn binding proteins datasets (Consortium, 2019). The results obtained from this search were shortlisted based on whether they are manually annotated, *i.e.*, from reviewed databases such as (Swiss-Prot) or computationally annotated, *i.e.*, unreviewed (TrEMBL). Only the reviewed (Swiss-Prot) proteins were taken. Furthermore, to reduce redundancy, the “UniRef100 database” was used to cluster the shortlisted reviewed proteins. The local databases of these selected reviewed Fe and Zn binding proteins from Uniprot were created using Basic Local Search Alignment Tool (BLAST) algorithm (Altschul et al., 1990).

### 2.2 Identification of iron and zinc-binding sequence motifs in the complete proteome of *Zea mays*

To identify Fe and Zn binding sequence motifs in the complete proteome of *Zea mays*, BLASTp search of the complete *Zea mays* proteome was done against the local databases of Fe and Zn at Expect value (e-value) of 0.00001. The proteins showed hits at e-value of less than or equal to 0.00001, and identity more than or equal to 50% was selected further for the second step. In the second step, the MetalPDB database was used to retrieve Fe and Zn binding protein datasets, and local BLASTp search of shortlisted proteins from the first step was further performed with these selected MetalPDB datasets at e-value of 0.00001 (Putignano et al., 2018). The proteins showed hits with MetalPDB datasets at e-value of less than or equal to 0.00001, and identity more than or equal to 50% was putatively considered to have Fe binding or Zn binding sequence motifs.

### 2.3 Identification of iron and zinc binding structural motifs

The selected Fe and Zn binding sequence motifs containing proteins were modeled by Protein Homology/analogY Recognition Engine V 2.0 (Phyre2) (Kelley et al., 2015). Further, the proteins were shortlisted manually based on modeling confidence, query coverage, and identity of more than or equal to 90, 50, and 30 percent, respectively. Energy minimization of these modeled proteins was performed using GROningen MAchine for Chemical Simulations (GROMACS) (Abraham et al., 2015). Further, to predict the structural Fe and Zn binding motifs, the PYMOL server was used to perform the structural alignment of Fe and Zn binding energy-minimized modeled proteins based on the templates obtained from the MetalPDB BLASTp search (DeLano, 2002).

### 2.4 Functional annotation, subcellular localization prediction and gene ontology analysis of putative Fe and Zn binding proteins of *Zea mays*

The bioinformatics servers Pfam, InterProScan, and NCBI-CDD were used for the functional annotation of shortlisted putative Fe and Zn binding proteins of *Zea mays* (Jones et al., 2014; Marchler-Bauer et al., 2015; Finn et al., 2016). These tools help to predict functional domains/families and further, the literature survey of predicted domains/families was performed to support the broad classification of shortlisted Fe and Zn binding proteins. Subcellular localization identification aids in determining the functional role of proteins. Therefore, cellular compartmentalization of putative Fe and Zn binding proteins of *Zea mays* was also done using consensus of three tools, i.e., LocTree3, pLoc-mPlant, and BUSCA (Goldberg et al., 2014; Cheng et al., 2017; Savojardo et al., 2018). Gene Ontology (GO) is the formal computational representation of biological systems that provide a set of concepts for explaining the function of genes and gene products from all organisms (Ashburner et al., 2000). A Cytoscape plugin ClueGO was used for Gene ontology prediction of predicted Fe and Zn binding proteins (Shannon et al., 2003; Bindea et al., 2009). Both GO networks, i.e., GO biological process and GO molecular function were configured on a kappa score of more than or equal to 0.4.The network nodes are represented as circle which indicate particular GO biological or molecular terms. The connections between the GO terms are based on their association indicated by an edge.

## 3 Results and discussion

### 3.1 Iron and zinc binding sequence motifs containing proteins in the complete proteome of *Zea mays*

**Figure 1:**
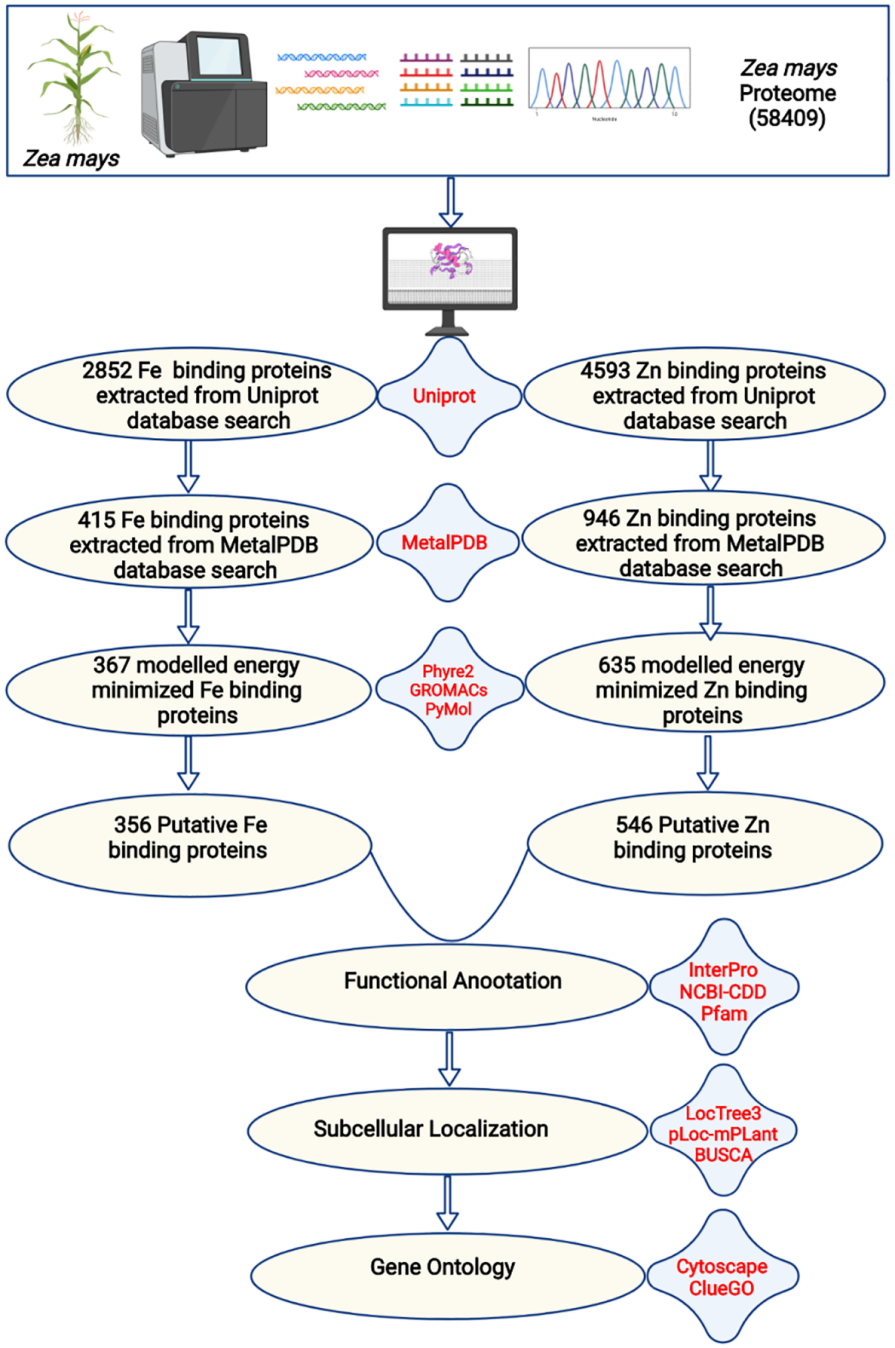
The complete description of the study. The complete proteome of *Zea mays* was extracted from the RefSeq ftp server of the National Centre for Biotechnology Information. The BlastP search of the complete proteome was done against iron and zinc-binding protein datasets from the UniProt database. The shortlisted proteins were searched against iron and zinc datasets of MetalPDB. Further, the selected proteins were modeled by Phyre2 and energy minimization was done by GROMACS. The structural alignment of the scrutinized proteins was done using PyMol to find out the putative metal (Fe or Zn) binding sites. A total of 356 Fe-binding and 546 Zn-binding proteins have been predicted from the complete proteome of *Zea mays*. These proteins were functionally characterized based on functional domains, subcellular localization, and gene ontology.

The complete proteome of *Zea mays* has a total of 58409 proteins. To search for Fe and Zn binding proteins among these 58409 proteins, both sequence- and structure-based search was performed. The complete description of the study is shown in **Figure 1**. The homology or sequence similarity search is the first, most informative, effective, and reliable step used in finding the regions of similarity or identity between two proteins which further articulate their structural, functional, and evolutionary features (Pearson, 2013). Generally, two proteins that share more than 30% identity during homology search are thought to be similar in their structure and function (Pearson, 2013). In the first step of this study, BLASTp search of 58409 *Zea mays* proteins was done against the local database of selected reviewed Fe and Zn binding proteins from Uniprot at e-value of 0.00001 and identity ≥ 50. A total of 2852 and 4593 proteins showed hits with Fe and Zn databases, respectively, at e-value less than or equal to 0.00001 and identity more than or equal to 50%. Further, to reduce false optimistic predictions, the second check was also performed in which a local BLASTp search of shortlisted proteins was performed against the Fe and Zn datasets of the MetalPDB database. Among 2852 proteins, 415 proteins showed hits with Fe binding proteins, and out of 4593 proteins, 946 proteins showed hits with Zn binding proteins from the MetalPDB database at e-value less than 0.00001 and identity more than or equal to 50%. These 415 and 946 proteins were putatively considered to have Fe and Zn binding sequence motifs, respectively.

### 3.2 Iron and zinc binding structural motifs containing proteins in *Zea mays*

**Figure 2:**
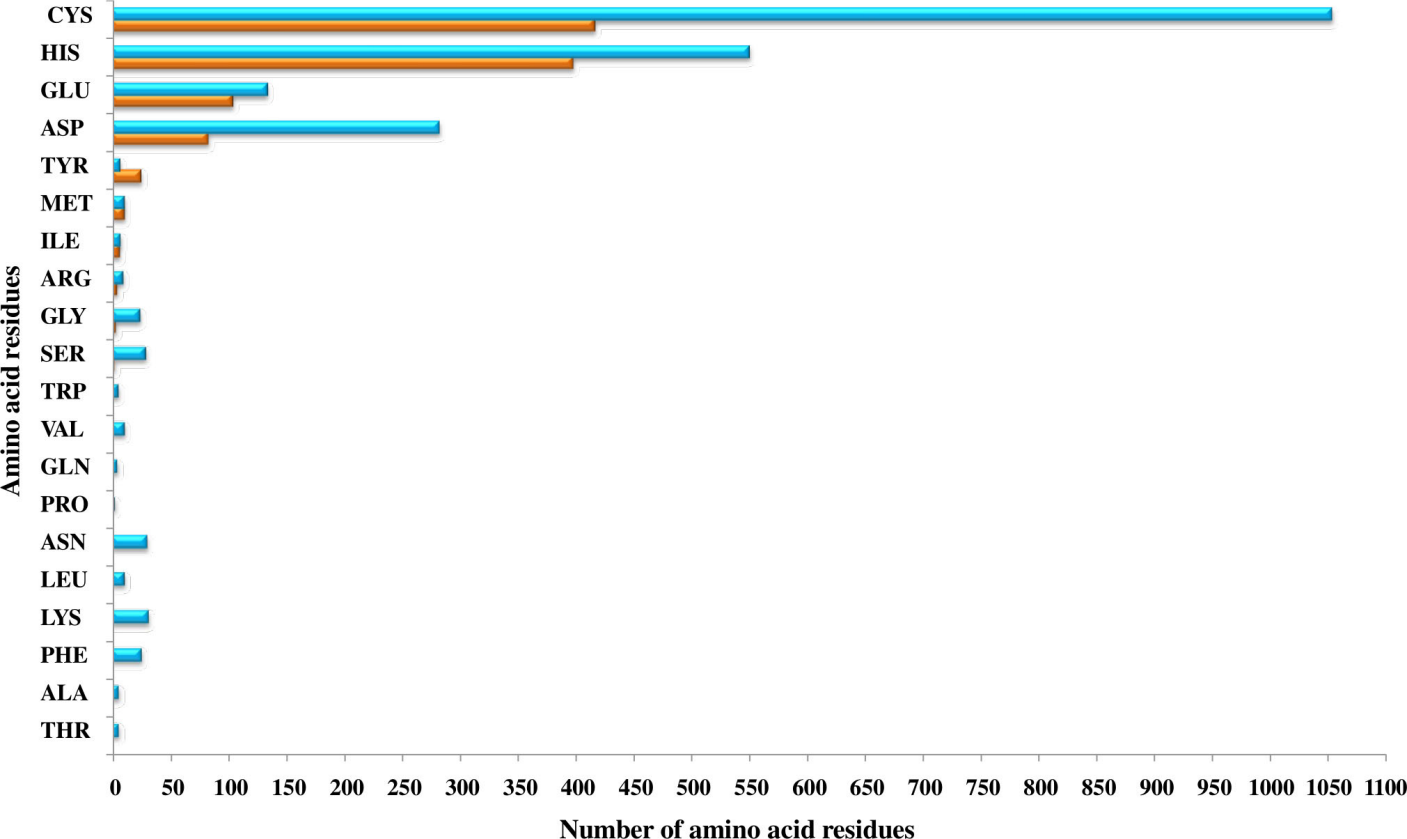
The pattern of amino acid residues in Fe- and Zn-binding proteins of *Zea mays*. Here, the X and Y axis indicate the number and name of amino acid residues present in the binding sites of putative Fe- and Zn-binding proteins of *Zea mays*, respectively. Cys, His, Glu, and Asp amino acid residues were found dominant in predicted Fe- and Zn-binding proteins of *Zea mays*.

The primary protein sequences fold to form 3D structures. Most of the proteins that share similar sequences, opt similar structures (Rost, 1999; Kosloff and Kolodny, 2008; Pearson, 2013). Thus, the shortlisted 415 Fe and 946 Zn binding-sequence motifs containing proteins were modeled by Phyre2. Out of 415 Fe binding, 367 and out of 946 Zn binding, 635 proteins were shortlisted manually with modeling confidence, query coverage, and identity of more than or equal to 90, 50, and 30 percent, respectively (**Supplementary Tables 1A, B**). The energetic state of a protein is a key parameter in determining its stability. Therefore, performing energy minimization or finding the set of coordinates representing the protein in the state of minimum energy and maximum stability is critically important (Mackay et al., 1989; Gautam, 2020). Energy minimization of shortlisted 367 Fe and 635 Zn binding modeled proteins was performed using GROningen MAchine for Chemical Simulations (GROMACS). To predict structural Fe and Zn binding sites, the structural alignment of Fe (Fe ions (Fe^2+^, Fe^3+^), heme, and Fe sulfur cluster) and Zn binding energy minimized modeled proteins was performed using PYMOL based on the templates obtained from MetalPDB BLASTp search. A total of 356 proteins out of 367 energy-minimized modeled proteins were found to have both Fe binding sequence and structural motifs. Moreover, Fe-containing proteins exists in three primary forms, i.e., free iron binding (Fe^2+^ and Fe^3+^), iron-sulfur cluster, and heme binding (Kaplan and Ward, 2013). Among these 356 proteins which were selected as putative Fe binding proteins, 135 proteins have free Fe ions (Fe^2+^ and Fe^3+^) binding sites, 127 have iron-sulfur cluster sites, and 134 have heme binding sites (134) (**Supplementary Table 2A**). Secondly, 546 proteins among 635 energy-minimized modeled proteins were found to have both Zn binding sequence and structural motifs (**Supplementary Table 2B**). Further analysis revealed that a total of 31 proteins shared both Fe and Zn binding motifs (**Supplementary Table 2C**) which are concerned with a previously known fact that some proteins have promiscuous reactivity interaction with more than one metal ion (Eom and Song, 2019). Cystine, histidine, glutamic acid, and aspartic acid residues are commonly found in the binding pocket of these putative Fe and Zn binding proteins. The pattern of interacting residues was in accordance with earlier studies which symbolize that transition metal ions are usually bound to polar and negatively charged residues (Cao et al., 2017). The pattern of interacting residues in the binding pockets of predicted Fe and Zn binding proteins of *Zea mays* is shown in **Figure 2**.

### 3.3 Functional domain annotation, subcellular localization, and gene ontology analysis of Fe binding proteins of *Zea mays*

**Figure 3:**
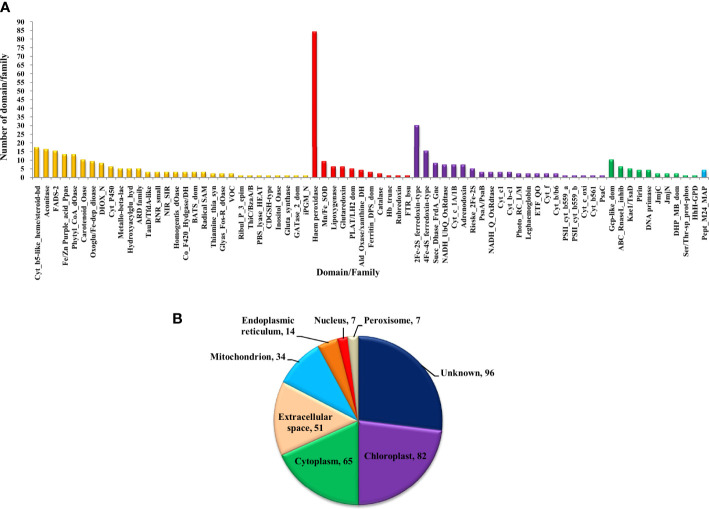
**(A)** Functional domain annotation of Fe-binding proteins of *Zea mays*. Here, the X-axis represents the name of a particular domain/family and Y-axis indicates the number of proteins having the particular domain/family. The color of a particular column represents its broad functional class. The Fe-binding proteins of *Zea mays* are divided into five broad functional classes, *i.e*., metabolism (yellow), stress response (red), transport (purple), gene expression and regulation (green), and proteolysis (sky-blue). The dominant classes were metabolism, stress response, and transport. The Iron proteome of *Zea mays* is found to be enriched in domains: Heme peroxidase, 2Fe-2S and 4Fe-4S ferredoxin-type, Cytochrome subunits b and c, Cyt b5-like heme/steroid-bd, Aconitase, Fatty acid desaturase type 2, Phytanoyl-CoA dioxygenase, Fe/Zn purple acid phosphatase, Carotenoid oxygenase, Gcp-like domain, Oxoglutarate/iron-dependent dioxygenase, Non-haem dioxygenase, Mn/Fe superoxide dismutase, Succinate dehydrogenase/fumarate reductase, and NADH. **(B)** Subcellular localization of Fe binding proteins of *Zea mays*. Here, the pie chart indicates the subcellular localization of Fe-binding proteins of *Zea mays*. The cellular localization of nearly 27% of proteins remains unidentified and approximately 73% of proteins are predicted to be present in seven different locations. The subcellular compartments: chloroplast (23%), cytoplasm (18%), and extracellular space (14%) are enriched in Fe-binding proteins followed by mitochondria (10%), endoplasmic reticulum (4%), nucleus (2%), and peroxisome (2%).

**Figure 4:**
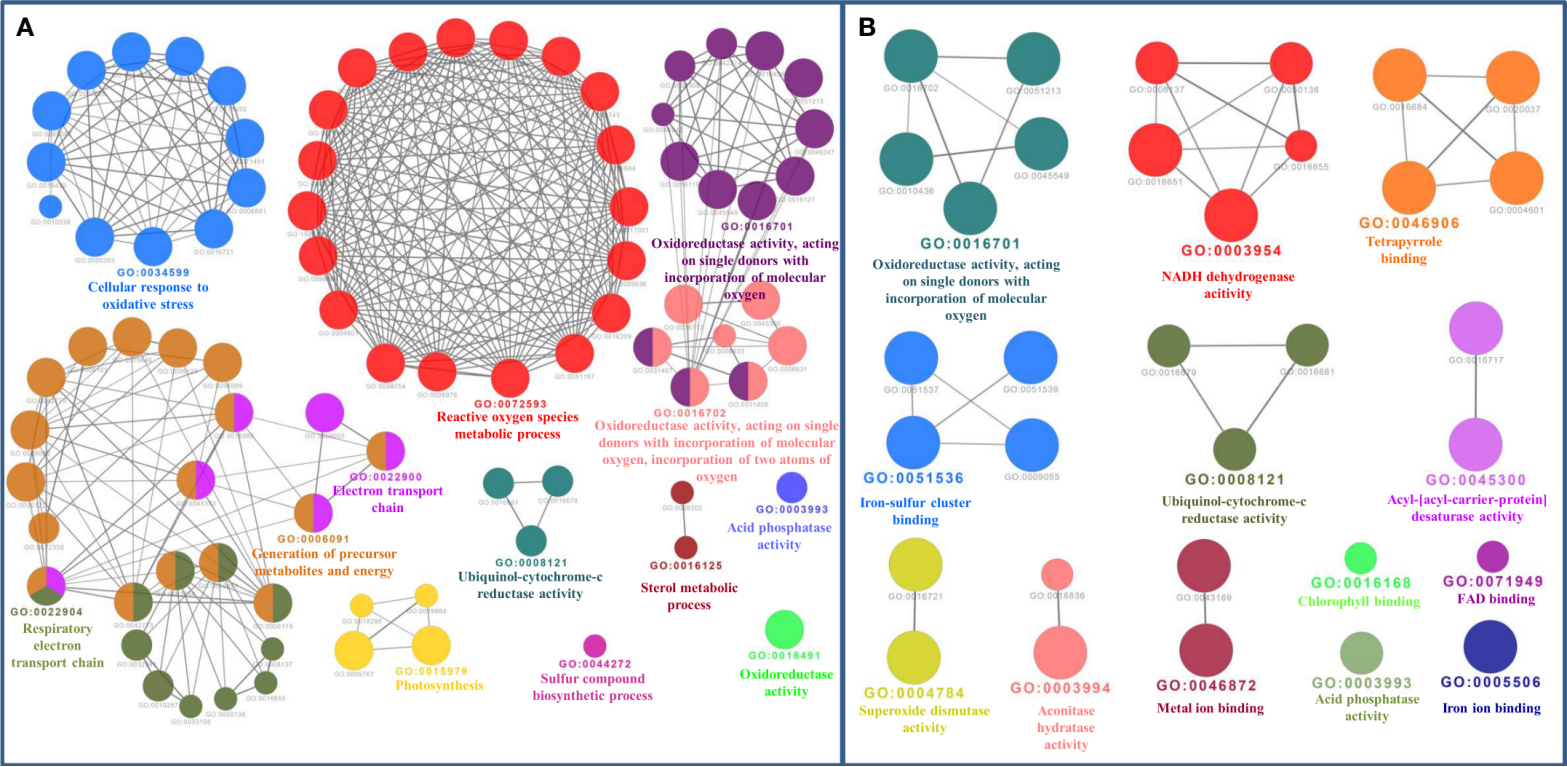
**(A)** Gene Ontology Biological Process Network of Fe-binding proteins of *Zea mays.* The biological process network was configured by the Cytoscape plugin ClueGO at a Kappa score ≥ 0.4. The network consists of 13 significant groups. Here, each circle represents the particular node or GO Biological Process term. The color of the node symbolizes the particular group they belong. The mixed color of the node specifies that the node belongs to multiple groups. **(B)** Gene Ontology Molecular Function Network of Fe-binding proteins of *Zea mays*. The Molecular Function Network was built on a Kappa score ≥ 0.4. The network has 13 significant groups. Each circle corresponds to the particular node or GO Molecular Function term. The color of the node signifies the particular group they belong and the mixed color of the node represents that the node belongs to multiple groups. Both networks are in accordance with the results of functional domain classification.

The bioinformatics servers Pfam and InterProScan were used for the functional annotation of Fe and Zn binding proteins of *Zea mays.* Heme peroxidase was the most predominant domain in predicted Fe binding proteins of *Zea mays*. 2Fe-2S and 4Fe-4S ferredoxin-type, cytochrome subunits b and c, Cyt b5-like heme/steroid-bd, aconitase, fatty acid desaturase type 2 (FADS2), Phytanoyl-CoA dioxygenase, Fe/Zn purple acid phosphatase (Fe/Zn PAP), carotenoid oxygenase, Gcp-like domain, oxoglutarate/iron-dependent dioxygenase, Non-haem dioxygenase (DIOX), Mn/Fe superoxide dismutase (Mn/Fe SOD), succinate dehydrogenase/fumarate reductase (SDR), and NADH: ubiquinone oxidoreductase were other common domains found in Fe binding proteins of *Zea mays*. Furthermore, based on the literature analysis of predicted domains or families in Fe-binding proteins, the Fe proteome of *Zea mays* was divided into five broad functional categories, namely, metabolism, stress response, transport, gene expression and regulation, and proteolysis. The dominant categories among them were metabolism, stress response, and transport. A detailed description of the functional domains among Fe-binding proteins of *Zea mays* and their probable functions based on the literature survey is given in **Supplementary Table 3A** and **Figure 3A**. Further, we were able to predict the subcellular localization of approximately 73% of the putative Fe-binding proteins. The predicted Fe-binding proteins of *Zea mays* were subcellularly found to be localized in seven different localizations. Among them, most of the proteins were found in chloroplast (23%), cytoplasm (18%), extracellular space (14%), and mitochondrion (9.6%) (**Supplementary Table 3A** and **Figure 3B**). The subcellular localization signifies the results of functional domains analysis as most of the Fe-binding proteins functional in metabolism was found to be localized in chloroplast andcytoplasm. The Fe-binding proteins involved in the transport of electrons were confined to mitochondria and chloroplast which is also in line with the findings of previous studies (Lu, 2018). Heme peroxidases proteins of *Zea mays* were subcellularly found to be constrained in extracellular space, cytoplasm, and peroxisome. Moreover, the GO biological process network of predicted Fe-binding proteins of *Zea mays* was constructed on 13 Kappa score groups having 84 GO biological process terms and 230 GO connections (**Supplementary Table 4A** and **Figure 4A**). All these 13 groups were found to be significant in the network. The GO molecular function network was also built on 13 Kappa score groups with 33 GO molecular terms and 27 GO connections (**Supplementary Table 4B** and **Figure 4B**). Both GO biological and molecular networks are in accordance with a functional classification which states that most of the predicted Fe-binding proteins of *Zea mays* are involved in metabolic processes of energy generation, transport of electrons, and response to stress.

The detailed analysis of Fe-binding proteins of *Zea mays* presented evidence that the dominant functional categories among them were metabolism, stress response, and transport. We have placed the proteins involved in the metabolic processes of carbohydrates, amino acids, fatty acids, nucleotides, secondary metabolites, energy generation, and destruction in the metabolism category. Cyt b5-like heme/steroid-bd, aconitase, FADS2, Fe/Zn PAP, Phytyl-CoA dioxygenase, carotenoid oxygenase, oxoglutarate/iron-dependent dioxygenase (Fe/2OG), and Cyt P450 were common domains listed in the category of metabolism. Studies on Cyt b5-like proteins in flowering plants provided evidence that these are heme-binding proteins that are localized to the endoplasmic membrane, act as redox components, and function as an obligate electron shuttle in fatty acid hydroxylation, sterol desaturation, hydroxylation, desaturation of sphingolipid long-chain base, and Cyt P450-mediated reactions to control lipid, sterol, and lignin metabolism (Kumar et al., 2012; Gou et al., 2019). Ikeyama et al. (2010) reported a cytoplasmic Cyt b5-like heme/steroid-bd containing protein RLF in arabidopsis which controls early cell division involved in lateral root formation and plays an indispensable role in root architecture (Ikeyama et al., 2010). The role of iron-sulfur containing aconitase proteins of land plants were earlier noticed in the TCA cycle for the reversible isomerization of citrate to isocitrate (Wang et al., 2016). A study on the repression of tobacco aconitase by nitric oxide suggested that it has drastic effects on photosynthesis and fruit yield (Moeder et al., 2007). Additionally, plant aconitases are also known to mediate other physiological processes like oxidative stress, lipid metabolism, glyoxylate cycle, sucrose metabolism, and the regulation of cell death (Wang et al., 2016). In previous studies, it is stated that FADS enzymes contain conserved histidine-rich sequences in their active sites as is present in verified divalent iron enzymes and catalyzes the desaturation of fatty acids in various forms which help in the regulation of fatty acid composition and maintenance of membrane fluidity under abiotic and biotic stress conditions (Zhao et al., 2019; Hajiahmadi et al., 2020). The PAPs enzymes require binuclear chromophoric metal ions (Fe/Zn or Fe/Mn) for their activity and are known to play vital roles in the maintenance of Pi acquisition and utilization, cellulose and carbon metabolism, fixation of nitrogen, reactive oxygen species (ROS) metabolism, response to nutrients, and other environmental stresses and pathogen defences (Olczak et al., 2003).

The non-heme iron enzymes carotenoid cleavage dioxygenases (CCDs) are known to reside in different subcellular compartments (cytoplasm or chloroplast) and play diverse roles in the biosynthesis of various secondary metabolites and the production of phytohormones, antioxidant, volatile and signaling molecules, pigments, flavors, aromas, and defense compounds (Dhar et al., 2020). Earlier, the potential role of the CCD1 gene in maize is suggested to be responsible for the targeting and regulation of provitamin-A content to combat global vitamin A deficiency (Vallabhaneni et al., 2010). Previous reports also suggested that higher plants’ genomes are rich in dioxygenases, mainly Fe/2OG and CytP450 (Mitchell and Weng, 2019). The non-heme Fe/2OGs comprise one of the large dioxygenase superfamily in higher plants to catalyze reactions like the hydroxylation, dealkylation, desaturation, epimerization, halogenations, cyclization, epoxidation, formation of peroxide, and ring expansion or contraction as well as play vital roles in the biosynthesis of various primary and secondary metabolites, DNA repair, histone, and cell wall protein modifications (Mitchell and Weng, 2019). The Fe-dependent Phytyl-CoA dioxygenase which is also called phytanoyl-CoA hydrolase is known to catalyze the α-hydroxylation of phytanoyl-CoA to α-hydrooxyphytanoyl-CoA in the α-oxidation pathway of phytanoyl degradation (Araújo et al., 2011). The role of CytP450 hemoproteins is well elucidated in the biosynthesis of secondary metabolites, fatty acid metabolism, hormone regulation, xenobiotic metabolism, antioxidant biosynthesis, and plant stress responses (Mitchell and Weng, 2019; Pandian et al., 2020). Further, it is well-stated that CytP450 enzymes are preferable candidates to engineer new abiotic and biotic stress-resilient crop species (Pandian et al., 2020).

Heme peroxidase was the prime domain in the second dominant category, i.e., the stress response of Fe-binding proteins of *Zea mays*. The study found a high number of class III peroxidases and a few class I haem peroxidases. The other common domains listed in the class of stress response were Mn/Fe SOD, Glutaredoxin, lipoxygenase, and PLAT/LH2. An earlier study documented that class III peroxidases are hemoproteins of the secretory pathway in higher plants that exist as large multigene families and are biomarkers for metallic stresses (Jouili et al., 2011). They play diverse roles in lignification, cell wall metabolism, shoot and root cell elongation, hormonal metabolism, fruit growth and ripening, ROS metabolism, plant defense, responses to hypoxia, abiotic and biotic stresses, wound healing, and seed germination *via* regulation of their peroxidative, hydrolytic, and ROS activities (Jouili et al., 2011; Shigeto and Tsutsumi, 2016; Hofmann et al., 2020). Further, class I peroxidases are also known to play a major role in the detoxification of ROS (Jouili et al., 2011). The role of Mn/Fe SOD proteins has been stated previously in superoxide metabolic processes, i.e., detoxification of ROS to respond to oxidative stresses (Shiriga et al., 2014; Stephenie et al., 2020). Moreover, the SOD proteins are already known as better target for improving human health (Shiriga et al., 2014; Stephenie et al., 2020). A previous study has illustrated that Fe-S-containing protein Glutaredoxins function in dynamic biological processes such as redox signaling, ROS and iron homeostasis, defense responses, hormone regulation, tolerance to salt, drought, temperature, heavy metals, and other abiotic and biotic stresses which make them a promising target for crop improvement to improve crop tolerance for various stresses (Wu et al., 2017). The non-heme lipoxygenases are extensively distributed in plants to catalyze hydroperoxidation of lipids containing cis-1,4-pentadiene and functions in various biological processes like seed germination, senescence, fruit ripening, and plant defense responses against biotic and abiotic stresses (Viswanath et al., 2020). Our study also found cytoplasmic lipoxygenases containing PLAT/LH2 domain. An earlier study elucidated that PLAT proteins could be attractive targets in crop improvement to raise abiotic stress tolerance without yield penalty. These proteins act as positive regulators of abiotic stress tolerance and plant growth (Hyun et al., 2014).

The transport category contains the proteins involved in the transport of electrons, ions, or any other molecules across the membrane. 2Fe-2S ferredoxin-type, 4Fe-4S ferredoxin-type, cytochrome subunits (Cytb6f, Cytb/b6, Cytb567, Cytc, Cytc 1A/1B, Cytbc1, and Cytf), NADH dehydrogenase subunits (NADH UqQ oxidoreductase 20, 55, and 75 and NADH quinone oxidoreductase chain I), SDR, and Rieske 2Fe-2S domain-containing proteins were commonly categorized in the transport class of Fe-binding proteins of *Zea mays*. It is a well-known fact that varying redox potential properties of Fe in Fe-S clusters play critical roles in the transport of electrons during various fundamental life-sustaining processes such as photosynthesis (in thylakoid membranes) and respiration (in the inner mitochondrial membrane) (Lu, 2018). In plants, Fe-S cluster proteins have been distributed in four subcellular localizations, i.e., chloroplast, mitochondria, nucleus, and cytoplasm (Lu, 2018). The 2Fe-2S ferredoxin-type iron-binding proteins function mainly during photosynthesis to transport electrons from Photosystem-I to ferredoxin NADP^+^ reductase for producing NADPH which is consumed during Co_2_ assimilation (Hanke and Mulo, 2013). Additionally, the role of plant ferredoxins is also noted in other metabolic processes such as chlorophyll, phytochrome and fatty acids production, nitrogen and sulfur assimilation, and maintenance of redox signaling and redox balance (Hanke and Mulo, 2013). The role of Photosystem-I (Fe-S) proteins (PsaA, PsaB, and PsaC) and Cytbf6 proteins having Riekse-type 2Fe-2S domain has also been reported in the transport of electrons during photosynthesis (Lu, 2018). Further, the function of Fe-S-containing proteins, NADH UqQ oxidoreductase subunits 20, 55, and 75, and NADH quinone oxidoreductase chain I (Complex-I), SDR (Complex-II) and Cytbc1 (Complex-III), is well described in respiratory electron transport (Lu, 2018). Moreover, NADH quinone oxidoreductase also acts as an antioxidant enzyme that protects plants from necrotrophic fungi and other oxidative stresses, i.e., helps maintain plant homeostasis regarding cellular redox states and defense signaling (Heyno et al., 2013). A type-II NADH quinone oxidoreductase is known to play a role in prenylquinone metabolism and vitamin K1 accumulation (Piller et al., 2011). A recent report on plant SDR provided evidence that SDR (Complex-II) acts as a key regulator of mitochondrial stress responses and cellular signaling as it influences the plant stress and defense responses, stomatal opening, early seeding establishment, and root elongation (Huang et al., 2019). The energy transducing enzymes Cytbc1 (Complex-III) contain Rieske Fe-S cluster which acts as proton-exiting gate are the central component of the respiratory electron chain, i.e., they help in the regulation of electron flow and maintenance of redox or reactive oxygen species signaling (Sarewicz et al., 2021).

A few numbers of proteins were found in the classes of gene expression and regulation (Gcp-like domain, Kae1/TsaD domain, and ABC RnaseL inhibitor) and proteolysis (Peptidase M24 methionine aminopeptidase). The role of Gcp and Kae1/TsaD proteins is stated earlier in the development of the N6-threonylcarbamoyl group on adenosine at positions 37 (t6A37), i.e., in tRNA threonylcarbamoyladenosine modification (Thiaville et al., 2014). The Fe-S cluster-containing protein ABC RnaseL inhibitor having ribosome dissociation activity is well known as an endogenous suppresser of RNA silencing in plants and plays critical roles in ribosome biogenesis, ribosome recycling, and translation termination (Navarro-Quiles et al., 2018). The cytoplasmic proteins Peptidase M24 methionine aminopeptidase were listed in the proteolysis category. Previously, the role of M24 has been stated in N-terminal methionine removal which significantly contributes to post-translational processes involved in plant growth and development (Ghifari et al., 2019). Hence, these findings showed that scrutinized Fe-binding proteins are critically required by *Zea mays* for proper growth, development, and survival.

### 3.4 Functional domain annotation, subcellular localization, and gene ontology analysis of Zn-binding proteins of *Zea mays*

**Figure 5:**
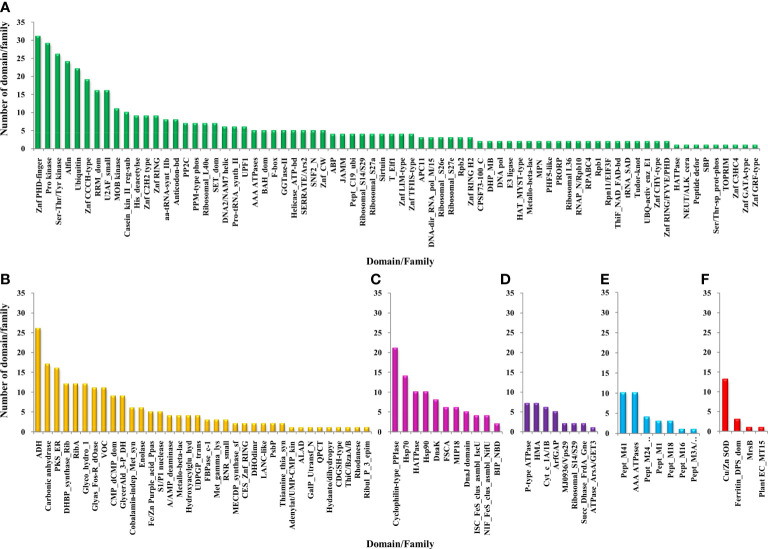
Functional domain annotation of Zn-binding proteins of *Zea mays*. Here, six-column graphs indicate the different broad functional classes of Zn-binding proteins of *Zea mays, i.e*., **(A)** Gene expression and regulation (green), **(B)** metabolism (yellow), **(C)** protein folding (pink), **(D)** transport (purple), **(E)** proteolysis (sky-blue), and **(F)** stress response (red) Here, the X-axis represents the name of a particular domain/family and the Y-axis indicates the number of proteins having the particular domain/family. The color of the particular column represents its broad functional class. The Zinc proteome of *Zea mays* is found to be enriched in Zinc finger transcription factors, Protein kinases, Alcohol dehydrogenase, Alfin, Ubiquitin, Cyclophilin-type PPIase, Carbonic anhydrase, Peptidase M41, and Cu/Zn SOD.

**Figure 6:**
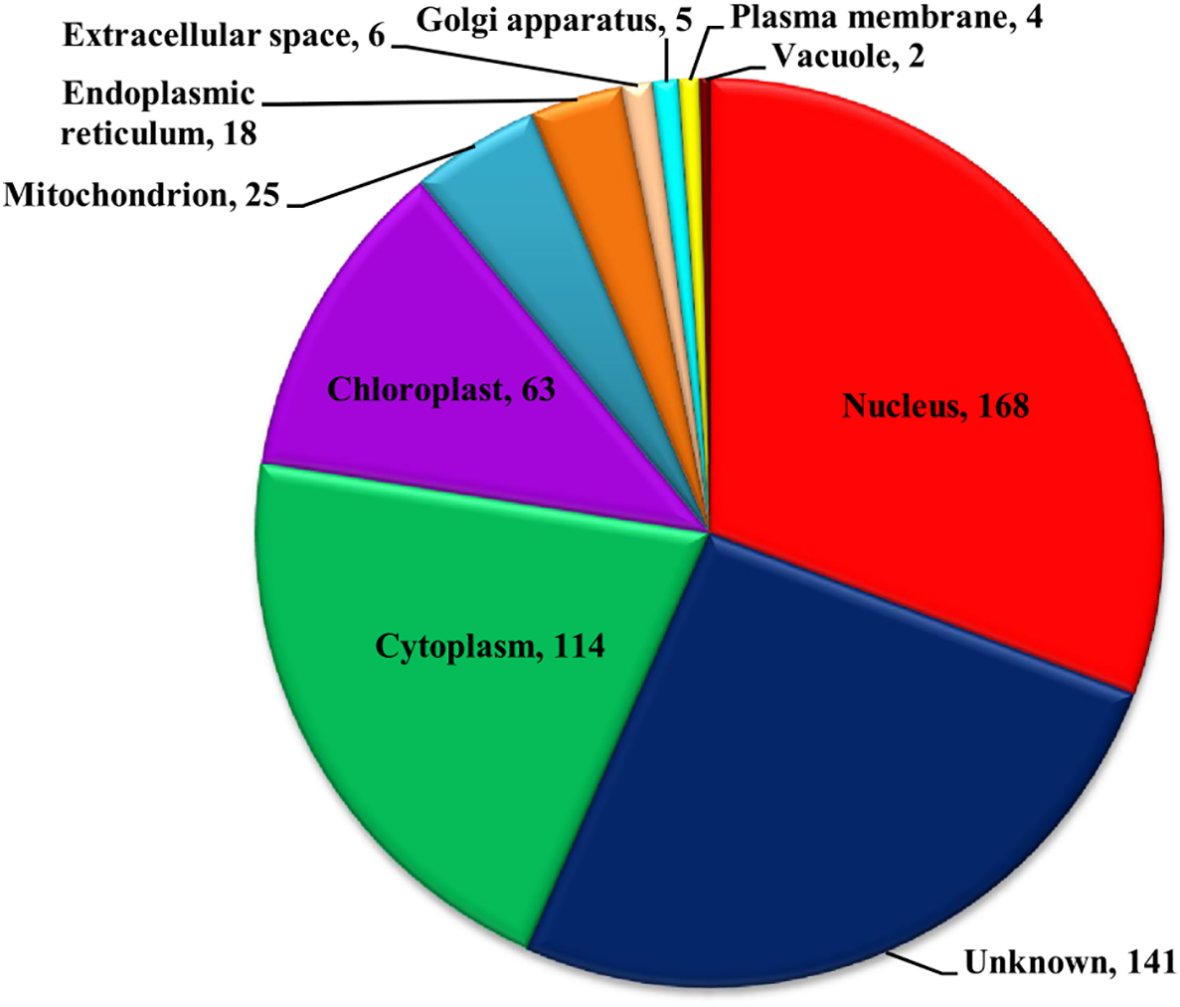
Subcellular localization of Zn-binding proteins of *Zea mays*. Here, the pie chart represents the cellular compartmentalization of Zn-binding proteins of *Zea mays.* The subcellular localization of approximately 26% of Zn-binding proteins of *Zea mays* remained unknown and nearly 74% of proteins were found to be localized in nine different subcellular compartments. Most of the Zn-binding proteins localized in the nucleus (31%), cytoplasm (21%), and chloroplast (12%).

**Figure 7:**
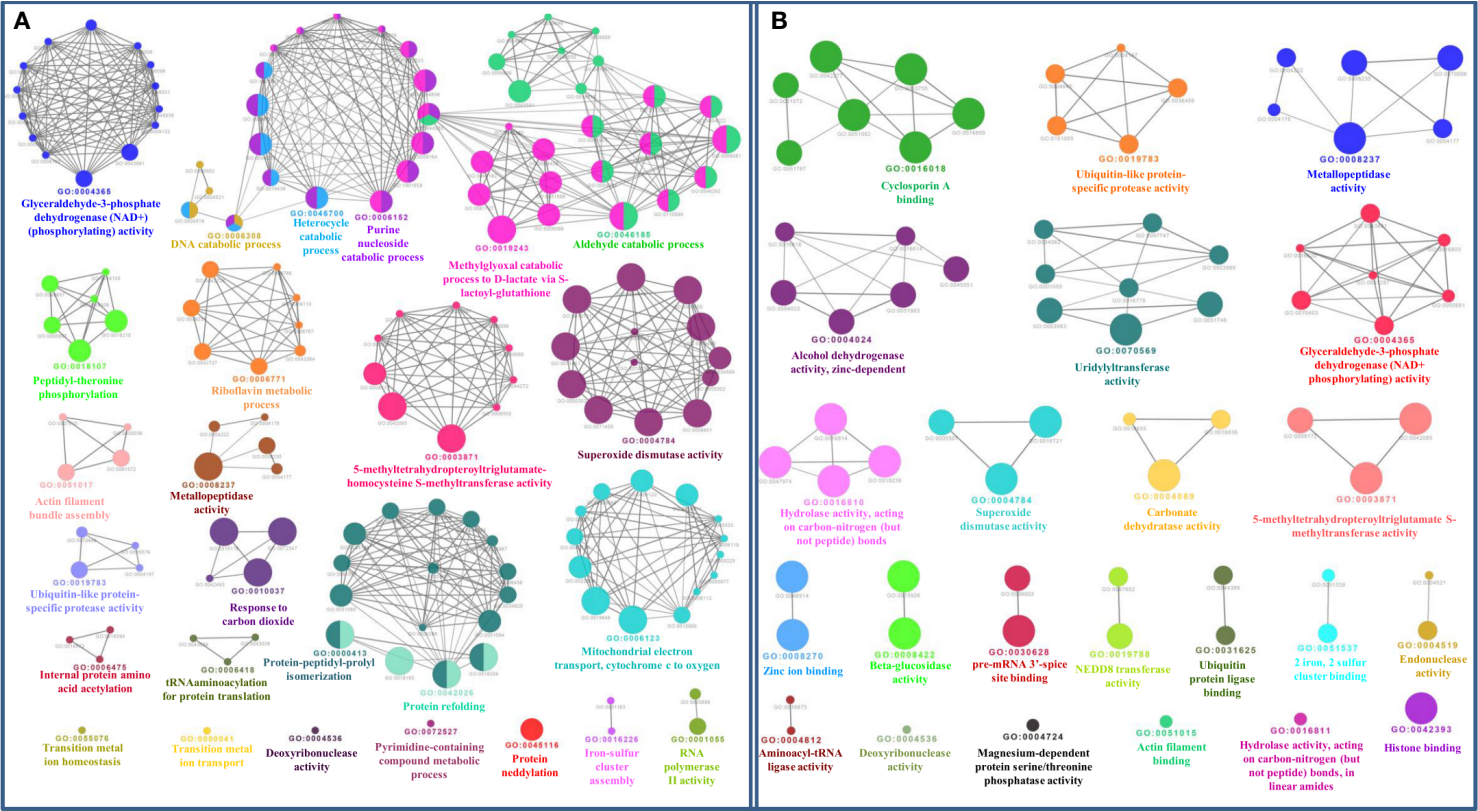
**(A)** Gene ontology biological process network of Zn-binding proteins of *Zea mays.* The Biological process network was constructed using the Cytoscape plugin ClueGO at a Kappa score ≥ 0.4. The network consists of 26 significant groups. The node or GO Biological Process term of the network was indicated in the form of a circle. The node color symbolizes the particular group they belong and the mixed color of the node specifies that the node belongs to multiple groups. **(B)** Gene ontology molecular function network of Zn-binding proteins of *Zea mays*. The Molecular Function Network was configured on the Kappa score ≥ 0.4. The network has 23 significant groups. Here, each circle signifies the particular node or GO Molecular Function term. The node color corresponds to the particular group they belong and the mixed color of the node indicates that the node belongs to multiple groups. Both networks support the findings of functional domain classification.

The functional domain analysis of Zn-binding proteins listed that Zinc proteome of *Zea mays* was enriched in Zinc finger transcription factors (PHD finger, CCCH-type, Ring, C2H2-type, CW, TFIIS-type, LIM-type, Ring-H2, Znf-Chy, GRF-type, GATA-type, and C3HC4) and Protein kinases (Ser-Thr/Tyr kinase), Alcohol dehydrogenase, Alfin, Ubiquitin, Cyclophilin-type PPIase, and carbonic anhydrase). Moreover, the broad classification based study of domains/families, study the Zn proteome of Zea mays was categorized into six classes, i.e., gene expression and regulation, metabolism, protein folding, proteolysis, stress response, and transport. The detailed description of the functional domains among Zn-binding proteins of *Zea mays* and their possible functions based on literature are listed in **Supplementary Table 3B** and **Figure 5**. Moreover, the predicted Zn-binding proteins of *Zea mays* were subcellularly found to be localized in nine different localizations. Among them, most of the proteins were found in the nucleus (31%), cytoplasm (21%), chloroplast (12%), mitochondrion (5%), and endoplasmic reticulum (3%) (**Supplementary Table 3B** and **Figure 6**). The subcellular localization signifies the results of functional domain analysis as most of the proteins functional in DNA metabolism are localized in the nucleus. The proteins involved in transcription, translation, and other metabolic activities are putatively localized in the cytoplasm. Further, the GO biological process network of putative Zn-binding proteins was constructed on 26 Kappa score groups having 156 GO biological process terms and 396 GO connections (**Supplementary Table 5A** and **Figure 7A**). The GO molecular function network was also built on 23 Kappa score groups with 73 GO molecular terms and 72 GO connections (**Supplementary Table 5B** and **Figure 7B**). Both GO biological and molecular networks are in accordance with functional classification which state that most of the predicted Zn-binding proteins of *Zea mays* are involved in gene regulation, metabolism, and protein folding activities.

The detailed analysis of Zn-binding proteins of *Zea mays* presented evidence that the dominant functional categories among them were gene expression and regulation, metabolism, and protein folding. Most of the Zn-binding proteins functionally involved in DNA replication, transcription, translation, RNA processing, post-transcriptional and post-translational modification, and regulation of cell cycle and cell signaling were categorized in the gene expression and regulation category. The common domain listed in this class were Zinc finger proteins (Znf PHD finger, Znf CCCH-type, Znf ring, Znf C2H2-type, Znf CW, Znf TFIIS-type, Znf LIM-type, Znf CHY-type, Znf GRF-type, Znf GATA-type, and Znf C3HC4), Alfin, Ubiquitin, Protein kinases (Ser-Thr/Tyr kinase, casein kinase II regulatory subunit, and MOB kinase activator), U2 auxiliary factor small subunit (U2AF), RNA recognition motif (RRM), Histone deacetylase, amino acyl-tRNA synthetase class-II, anticodon binding, ribosomal subunits (S14/29, S26e, S27a, S27e, L36, and L40e), SET domain, and PPM-type protein phosphatase 2C family (PP2C). Several types of zinc finger proteins are well described in the literature as transcription factors that contain “finger” domains rich in cysteine and histidine and play crucial roles in plant growth and development, stress resistance, and signal transduction (Peng et al., 2012; Wang et al., 2015; Sun et al., 2019). A report on maize Znf PHD finger proteins listed formerly that the majority of these proteins localized in the nucleus and regulates transcription, chromatin structure, and responses to multiple stresses (Wang et al., 2015). The Znf CCCH-type proteins are RNA-binding proteins that perform regulatory functions in mRNA processing, flower development, embryogenesis, tolerance to salt, drought, pathogens, and other abiotic and biotic stresses (Peng et al., 2012). The functions of Znf ring proteins are well documented previously in the regulation of plant development processes such as photomorphogenesis, flowering time, development of floral organs, roots, nodules, leaves, and fruits, and plant stress resistance (Sun et al., 2019). Znf LIM-type proteins play vital roles in the transcription regulation of biological pathways, determination of organ size, actin dynamics, pollen and fiber development, and plant immunity (Srivastava and Verma, 2017). Znf GRF-type proteins regulate stem elongation, cell expansion in leaf and cotyledon tissues, and stress responses (Huang et al., 2021). The Znf GATA-type proteins are known to regulate flowering time and flower development, leaf growth, photoperiodism, and optical signal transduction (He et al., 2018). Thus, the members of the Znf family have the potential to act as appropriate targets for the improvement of desired crop features. An earlier study documented that the U2AF small subunit localized in the nucleus contains RRM and two CCCH zinc fingers domains and plays essential roles in mRNA splicing *via* splicesome which regulates flowering time, leaf morphology, and flower and silique shape in plants (Wang and Brendel, 2006; Park et al., 2019). The Alfin proteins are also crucial in abiotic and biotic stresses (Zhou et al., 2017). The ubiquitin proteins of plants help in protein binding and regulation of protein stability, cellular processes, and signaling pathways which play critical roles in mounting a coordinated response to various environmental stresses (Miricescu et al., 2018).

The kinase proteins are known earlier to catalyze protein phosphorylation which plays a vital role in signal transduction pathways involved in growth, metabolism, differentiation, cell death and responses to light, pathogens, hormones, nutrient deprivation, and abiotic and biotic stresses (Stone and Walker, 1995; Chou et al., 2004; Jia et al., 2020). A study has also stated that the activity and localization of the Ser-Thr/Tyr kinases have been implicated in response to intracellular concentration (Chou et al., 2004; Jia et al., 2020). Jia et al. (2020) recently reported a Ser-Thr/Tyr kinase-encoding gene Kernal Number Row 6 which determines pistillate floret number ear length and regulates maize grain yield (Jia et al., 2020). Further, the casein kinase II, i.e., evolutionarily conserved Ser/Thr kinase is also known to require Zn-binding motifs for their functional activity and to influence several developmental stress-responsive pathways such as light signaling, circadian rhythm, regulation of replication, transcriptional, translational, recombination and cell cycle, response to abiotic and biotic stresses, flowering time, hormone responses, and DNA repair (Filhol et al., 2005; Mulekar and Huq, 2014). The MOB kinase activator proteins are known as regulators of kinases that function in appropriate plant development, i.e., correct patterning of the root tip, control of cell division, cell proliferation, cell differentiation and programmed cell death, and regulation of plant root growth in response to abiotic and biotic stresses (Pinosa et al., 2013). Moreover, the PPM2C proteins catalyze protein dephosphorylation and act as key players in signaling pathways involved in the regulation of plant responses to stresses and plant development (Singh et al., 2016). Hence, kinases and phosphatase proteins may act as suitable targets for crop improvement.

In the category of metabolism, the ADH, Polyketide synthase enoyl reductase (PKS-ER), CA, RibA, DHBP synthase RibB, and glycoside hydrolase 1 domain were noticed frequently. The plant ADHs encoded by the multigene family act as molecular markers and catalyze the interconversion of acetaldehyde to ethanol and other short linear alcohols/acetaldehyde pairs using Zn as a cofactor (Thompson et al., 2010). Plant ADHs are functionally diverse and play essential roles in various growth, development, and adaptation processes such as anaerobic metabolism, pollen and seedling development, fruit ripening, ROS homeostasis, and response to different abiotic and biotic stresses like hypoxia, dehydration, cold, salt, drought, flood, temperature, and mechanical damage (Thompson et al., 2010; Su et al., 2020). Further, the role of PKS that belongs to the medium-chain dehydrogenase/reductase superfamily has also been listed in the biosynthesis of secondary metabolites ranging from different signaling molecules to bioactive natural products (Kwan and Leadlay, 2010). The Zn metalloenzymes CAs are well known to catalyze the interconversion of CO_2_ to bicarbonate (Kolbe et al., 2019). Recently, a report on maize CAs presented the evidence that CAs reside in different subcellular localization and serve diverse roles in photosynthesis, light-dependent development and photorespiration, carbon starvation including sugar signaling, amino acid biosynthesis, and lipid metabolism, and CO2 stomatal signaling pathway (Kolbe et al., 2019). A study previously stated that the ribA gene of Arabidopsis has bifunctional activities of both GTP cyclohydrolase II (RibA) and 3,4-dihydroxy-2-butanone (RibB) and requires Zn ion to catalyze the biosynthesis of vitamin B2 (riboflavin) which acts as a precursor for flavoenzymes (Fischer and Bacher, 2011). The enzymes of riboflavin biosynthesis pathways are necessarily required by plants, plant pathogens, and human pathogens; however, they are absent in humans and animals. Hence, they serve as promising candidates for designing novel herbicides, fungicides, and anti-infective drugs that could be free from target-related toxicity (Fischer and Bacher, 2011). The role of the plant multigene encoded family GH1 has been illustrated formerly in the hydrolysis of cell wall-derived oligosaccharides during germination, lignifications, defense against herbivores, activation of phytohormone levels, and response to abiotic and biotic stresses (Xu et al., 2004; Zhao et al., 2012).

In the class of protein folding Cyclophilin-type PPIase, HSP70 and HSP90 domains were commonly noticed. Cyclophilin-type PPIase is known to catalyze the cis-trans isomerization of the peptide bond preceding a Pro residue which is necessary for the correct assembly or folding of protein complexes. These enzymes are highly versatile, are found to be localized across all the cellular compartments, and perform multiple physiological functions in plants such as transcriptional regulation, organogenesis, determination of cell-polarity, embryogenesis, seed development, mRNA processing, photosynthetic and hormone signaling pathways, plant defenses, disease resistance, and responses to abiotic and biotic stresses (Singh et al., 2020). HSPs are ubiquitous molecular chaperones and are a crucial component in maintaining protein homeostasis under stress and normal cellular processes by forming versatile functional networks for protein folding, assembly, translocation, aggregation, maturation, stabilization, and degradation (Park and Seo, 2015). An earlier study illustrated very well that HSP70 and HSP90 are suitable targets to develop stress-resistant and tolerant crops because of their broad cellular distribution and indispensable roles in the wide spectrum of physiological processes ranging from plant growth to folding of protein kinases, transcription factors and hormone receptors, regulation of circadian clock, anterograde transport of proteins to the chloroplast, and development of stress signal transduction, i.e., response to environmental stresses, diseases, and pest (Park and Seo, 2015; di Donato and Geisler, 2019; Khan et al., 2019).

We have found that a small number of Zn-binding proteins are enlisted in the, proteolysis, and stress response categories. HMA P-type ATPase, Peptidase M41, and Cu/Zn SOD were prime proteins listed in transport, proteolysis, and stress response, respectively. It has been well established that heavy metal P-type ATPases function in the uptake, transportation, accumulation, distribution, and detoxification of metallic ions across the membranes using ATP and, hence, play a significant role in maintaining metal ion homeostasis (Zhiguo et al., 2018). Previously, it was stated that the peptidase M41 family is the group of FtsH proteins that contain an AAA ATPase domain and Zn-binding motifs, subcellularly restrict to chloroplast or mitochondria, and play crucial roles in the biogenesis of thylakoid membranes and PSI complex, D1 degradation in the PSII repair cycle, singlet oxygen and executer1-mediated retrograde signaling, degradation and assembly of photosynthetic electron transport pathways proteins, and regulation of thermomemory in plants (Kato and Sakamoto, 2018; Mishra et al., 2019). Thus, these findings showed that shortlisted Zn-binding proteins are critically required by *Zea mays* for proper growth, development, and survival.

## 4 Conclusion

Conclusively, a precise *in silico* approach has been used in the current study to predict the Fe and Zn proteome of *Zea mays*. From the complete proteome of maize, nearly 0.6% and 1% proteins were found to have both sequence and structural motifs for Fe and Zn metal ions, respectively. Based on functional domain annotation, subcellular localization, and gene ontology analysis, which was further supported by earlier known literature, the predicted Fe-binding proteins of maize were putatively found to be functionally enriched in metabolism, transport, and stress response categories. On the other side, gene expression and regulation, metabolism, and protein folding categories were found to be dominant in Zn-binding proteins of maize. Thus, the computationally scrutinized and annotated Fe and Zn-binding proteins may play versatile roles to regulate the growth, development, survival, and fitness of maize plants. Moreover, the shortlisted proteins here are novel targets for experimental characterization and validation and further probably act as promising candidates for various crop improvement programs.

## Data availability statement

The original contributions presented in the study are included in the article/**Supplementary Material**. Further inquiries can be directed to the corresponding authors.

## Author contributions

AS and SV: Conceptualization and Methodology; AS and DS: Data analysis and validation; AS: Writing-Original draft preparation; DS and SV: Editing and suggestions. All authors contributed to the article and approved the submitted version.
